# Safety and quality of life with maintenance olaparib plus bevacizumab in older patients with ovarian cancer: subgroup analysis of PAOLA‑1/ENGOT-ov25

**DOI:** 10.1093/oncolo/oyae322

**Published:** 2024-12-14

**Authors:** Coline Montégut, Claire Falandry, Saverio Cinieri, Claire Cropet, Laure Montane, Frédérique Rousseau, Florence Joly, Malak Moubarak, Anna M Mosconi, Eva M Guerra-Alía, Christian Schauer, Hiroyuki Fujiwara, Ignace Vergote, Gabriella Parma, Gabriel Lindahl, Amélie Anota, Ulrich Canzler, Frederik Marmé, Eric Pujade-Lauraine, Isabelle Ray-Coquard, Renaud Sabatier

**Affiliations:** Département d’Oncologie Médicale, Institut Paoli-Calmettes, Marseille, France; GINECO, France; GINECO, France; Hospices Civils de Lyon, Université Lyon 1, Lyon, France; U.O.C. Oncologia Medica, Ospedale Senatore Antonio Perrino, Puglia, Italy; MITO, Italy; Department of Clinical Research and Innovation, Centre Léon Bérard, Lyon, France; Department of Clinical Research and Innovation, Centre Léon Bérard, Lyon, France; Département d’Oncologie Médicale, Institut Paoli-Calmettes, Marseille, France; GINECO, France; GINECO, France; University Unicaen, Inserm U1086 Anticipe, Centre François Baclesse, Caen, France; Department of Gynecology & Gynecologic Oncology, Kliniken Essen-Mitte, Essen, Germany; MITO, Italy; S.C. di Oncologia Medica, Ospedaliera S. Maria della Misericordia, AO di Perugia, Perugia, Italy; Hospital Ramon y Cajal, Madrid, Spain; GEICO, Spain; Hospital Barmherzige Brüder Graz, Graz, Austria; AGO-Austria, Austria; Jichi Medical University, Tochigi, Japan; GOTIC, Japan; University Hospital Leuven, Leuven Cancer Institute, Leuven, Belgium; BGOG, Belgium; European Institute of Oncology IRCCS, Milan, Italy; MANGO, Italy; Department of Oncology, Linköping University Hospital, Linköping, Sweden; NSGO, Sweden; Department of Clinical Research and Innovation, Centre Léon Bérard, Lyon, France; Universitätsklinikum Carl Gustav Carus, Technische Universität Dresden, Dresden, Germany; National Center for Tumor Diseases (NCT), Dresden, Germany; AGO, Germany; AGO, Germany; Universitätsklinikum Heidelberg, Heidelberg, Germany; GINECO, France; ARCAGY Research, Paris, France; GINECO, France; University Unicaen, Inserm U1086 Anticipe, Centre François Baclesse, Caen, France; Centre Léon BERARD and University Claude Bernard Lyon I, Lyon, France; Département d’Oncologie Médicale, Institut Paoli-Calmettes, Marseille, France; GINECO, France

**Keywords:** ovarian cancer, safety, quality of life, elderly, geriatric assessment

## Abstract

**Background:**

In PAOLA-1/ENGOT-ov25, the addition of olaparib to bevacizumab maintenance improved overall survival in patients with newly diagnosed advanced ovarian cancer. We describe the safety profile and quality of life (QoL) of this combination in older patients in PAOLA-1.

**Methods:**

Safety (CTCAE v4.03) and QoL (EORTC QoL Questionnaires Core 30 and Ovarian 28) data were collected. We compared safety by age (≥70 vs <70 years) in the olaparib-containing arm. QoL by treatment arm was assessed in older patients. Geriatric features, including Geriatric Vulnerability Score (GVS), were also gathered.

**Results:**

Of 806 patients randomized, 142 were ≥70 years old (olaparib-containing arm: *n* = 104; placebo arm: *n* = 38). Older patients treated with olaparib exhibited a similar safety profile to younger patients, except for higher rates of all grades of lymphopenia and grade ≥3 hypertension (31.7% vs 21.6%, *P =*.032 and 26.9% vs 16.7%, *P =*.019, respectively). No hematological malignancy was reported. Two years after randomization, mean Global Health Status and cognitive functioning seemed better with olaparib than bevacizumab alone (adjusted mean difference: +4.47 points [95% CI, −0.49 to 9.42] and +4.82 [−0.57 to 10.21], respectively), and other QoL items were similar between arms. In the olaparib-containing arm, older patients with baseline GVS ≥ 1 (*n* = 48) exhibited increased toxicity and poorer QoL than those with GVS of 0 (*n* = 34).

**Conclusion:**

Among older patients in PAOLA-1, olaparib plus bevacizumab had a manageable safety profile and no adverse impact on QoL. Additional data are required to confirm these results in more vulnerable patients.

(ClinicalTrials.gov Identifier: NCT02477644).

**Implications for Practice**

Our objective was to describe safety and quality of life (QoL) with maintenance olaparib plus bevacizumab in older patients (≥70 years) in the phase III PAOLA-1 trial.

Compared with younger patients, older patients treated with the combination exhibited higher rates of all grades of lymphopenia and grade ≥3 hypertension. They did not exhibit hematological malignancy or treatment-related death. Within 2 years postrandomization, Global Health Status and cognitive functioning scores tended to be better and other QoL items were similar in the olaparib versus placebo arm.

Thus, selected older patients can benefit from this combination without major toxicity and QoL impairment.

## Introduction

Ovarian cancer (OC) is the leading cause of mortality among patients with gynecologic malignancies in Western countries. Because of increased life expectancy, almost half of all OC occurs in elderly women.^1^

Older age is one of the prognostic factors of OC with the poorest outcomes.^2^ However, increasing evidence indicates that chronological age, without consideration of clinical condition, should not adversely influence the ability of older patients to receive standard-of-care treatment.^3-6^ Given the variability in frailty and clinical condition in an older population, accounting for geriatric factors will help to improve their management. In line with this objective, the Geriatric Vulnerability Score (GVS) was developed by The French National Group of Investigators for the Study of Ovarian and Breast Cancer (GINECO). Based on geriatric covariates, this specific ovarian tool can help clinicians detect vulnerable older OC patients presenting a high risk of premature death and toxicity during chemotherapy.^7,8^ In the EWOC-1 trial, vulnerable patients, defined as having GVS ≥ 3, were randomized to have either a standard carboplatin–paclitaxel doublet, single-agent carboplatin, or a 3-/4-weekly carboplatin–paclitaxel doublet.^7,8^ Patients treated with carboplatin alone had poorer overall survival (OS), indicating that even the most vulnerable patients should not be excluded from standard treatment.

For decades, surgical cytoreduction and platinum–taxane doublet therapies have been the mainstay treatment of OC. The randomized phase III PAOLA-1/ENGOT-ov25 study (NCT02477644) demonstrated that the addition of the poly(ADP-ribose) polymerase (PARP) inhibitor olaparib to bevacizumab maintenance following standard platinum-based therapy plus bevacizumab improved progression-free survival (PFS) and OS in patients with newly diagnosed advanced OC with homologous recombination deficiency (HRD)-positive disease.^9,10^ In patients older than 65 years, a 5-month improvement in PFS (median 21.6 months with olaparib vs 16.6 months with placebo) in the intent-to-treat (ITT) population was seen, and PFS was greater with olaparib in patients with *BRCA1*/*BRCA2* mutation (BRCAm) and/or HRD-positive tumors.^11^ Moreover, several studies report that bevacizumab monotherapy yields similar effects in older OC patients despite a poorer safety profile than in younger patients.^12-16^ To the best of our knowledge, no prior geriatric assessments and quality of life (QoL) evaluations in older patients receiving olaparib have been reported.

Additionally, patient assessment of health-related QoL (HRQoL) is an important concern in older populations. Like survival, patient-reported outcomes are key determinants of treatment benefits.^17-19^

This preplanned, exploratory analysis of PAOLA-1 aimed to describe safety and HRQoL in older patients with advanced OC who were treated using first-line olaparib–bevacizumab maintenance therapy. We also explored geriatric features that may be associated with toxicity and HRQoL.

## Methods

### Study design and participants

PAOLA-1 was a randomized, double-blind, phase III trial and was conducted in 11 countries from July 2015 to September 2017. A total of 806 patients with newly diagnosed, histologically confirmed, advanced (International Federation of Gynecology and Obstetrics [FIGO] stage IIIB-IV) high-grade OC, primary peritoneal cancer, and/or fallopian tube cancer, who had no evidence of disease or an objective response following completion of first-line platinum–taxane–bevacizumab treatment, were randomly assigned (2:1 ratio) to receive olaparib (300 mg) or placebo twice daily for up to 24 months in combination with standard bevacizumab maintenance (up to a total of 15 months). The study design, inclusion/exclusion criteria, and primary endpoint (and prespecified analysis of outcomes by tumor BRCAm and/or HRD status) have already been published.^10^

For this preplanned exploratory endpoint of the PAOLA-1 trial, safety and HRQoL were analyzed and segregated according to patient age and treatment arm. The cutoff age defining older patients was 70 years at the time of diagnosis. First, the safety profile of older patients (≥70 years) treated with olaparib was described and compared with that of younger patients (<70 years). Second, we compared HRQoL in older patients between the olaparib and placebo arms. Finally, geriatric covariates were gathered to examine their predictive value on toxicity and HRQoL.

The study was approved by an international ethics committee, according to the provisions of the Declaration of Helsinki and Good Clinical Practice Guidelines, and all patients provided written informed consent.

### Data collection and assessments

For all patients, clinical status (assessed by Eastern Cooperative Oncology Group performance status [ECOG PS] score), FIGO stage, histology, and type of surgery were collected before randomization, and tumor BRCAm and HRD status were centrally assessed.^10^ Data included in these analyses were collected from the data cutoff in March 2019.

At baseline, specific geriatric covariates were gathered for patients aged ≥70 years. Nutritional status was assessed using body mass index (BMI) and albumin level. The definition of malnutrition was a BMI value of ≤21 kg/m^2^ and/or albumin levels of <35 g/L,^20^ and obesity was defined as a BMI value of >33 kg/m^2^.^21^ Polypharmacy was defined as ≥5 drugs/day, and comorbidities were assessed using the age-adjusted Charlson Comorbidity Index (CCI, severe score ≥ 5).^22-24^ Functional status was determined using the Activities of Daily Living (ADL) scale (impaired < 6)^25^ and Instrumental Activities of Daily Living (IADL) scale (impaired < 25).^26^ Depression was assessed using the Hospital Anxiety Depression Scale (HADS), with an impaired score of ≥ 14, which was validated in OC.^27^ Vulnerability was assessed using the GVS, which is the sum of 5 criteria, and ranges from 0 to 5 (1 point for each item; higher scores indicate more vulnerable patients): (1) albuminemia <35 g/L; (2) ADL score < 6; (3) IADL score < 25; (4) lymphopenia <1 G/L; and (5) HADS ≥ 14. GVS has initially been validated to assess cytotoxic chemotherapy toxicities in older OC populations.^28^ As no validated cutoff has been defined for the GVS in patients receiving PARP inhibitors, an a priori threshold was not set for this parameter. All clinical and biological parameters were prospectively collected before randomization.

Safety outcomes reported were dose reductions, treatment interruption, and discontinuation due to toxicity. Adverse events (AEs) were scored using the Common Toxicity Criteria for Adverse Events version 4.03 (CTCAE v4.03). Serious AEs of specific interest were monitored considering olaparib exposure (in particular myelodysplastic syndrome, acute leukemia, new primary cancer, and pneumonitis) and bevacizumab exposure (grade ≥3 hypertension, proteinuria, wound healing complications, hemorrhage, congestive heart failure and venous thromboembolic events, any-grade gastrointestinal perforation, abscesses, fistulae, arterial thromboembolic events, central nervous system bleeding and reversible posterior leukoencephalopathy syndrome, and grade ≥2 hemoptysis). Outcomes were analyzed in all randomly assigned patients who received at least 1 dose of the study drug.

For HRQoL analyses, patient-reported outcomes were assessed using 2 validated questionnaires. The European Organization for Research and Treatment of Cancer (EORTC) Quality of Life Questionnaire Core 30 (QLQ-C30) is a 30-question cancer-specific questionnaire that can be used to analyze 5 functional scales (physical, role [work and household activities], emotional, cognitive, and social), 9 symptom scales (fatigue, nausea and vomiting, pain, dyspnea, insomnia, appetite loss, constipation, diarrhea, and financial difficulties), and Global Health Status (GHS).^29^ The EORTC Quality-of-Life Questionnaire-Ovarian 28 (QLQ-OV28) is a specific OC module designed for patients with local or advanced disease who receive surgical treatment with or without chemotherapy.^30^ This questionnaire includes 28 items and allows the assessment of 3 functional (body image, attitude to disease and treatment, and sexuality) and 5 symptomatic scales (abdominal/gastrointestinal symptoms, peripheral neuropathy, hormonal/menopausal symptoms, hair loss, and other toxic effects of chemotherapy treatment). For both questionnaires, scores ranged from 0 to 100. Higher scores for GHS/QoL or functional scales indicate better function or QoL, whereas higher scores for symptom scales indicate increased severity of symptoms (poorer QoL). Outcomes were analyzed in all randomly assigned older patients (full analysis set). The QLQ-C30 and QLQ‑OV28 questionnaires were prospectively collected at baseline and then every 12 weeks for 2 years.

### Statistical analysis

Patient demographics, disease characteristics, and patterns of care at baseline were described by treatment arm and age category in terms of median and range for quantitative data and number and frequency for categorical data. This analysis contained data from 806 randomized patients (ITT population), including 802 patients receiving 1 or more doses of the study drug and 770 completing at least baseline HRQoL assessments.

AEs were described in terms of the number and frequency of patients who experienced each type of event at least once. Event frequencies were compared between age groups using a Chi-squared (χ^2^) test.

Compliance with HRQoL questionnaires was defined as the ratio of the number of patients having completed at least 1 item of the QLQ-C30 and QLQ-OV28 questionnaires to the number of patients remaining in the study at the theoretical time of questionnaire completion. The minimally important difference was defined to quantify the clinically relevant change or difference between treatment arms. Postbaseline scores or items that changed more than 4 points were clinically significant.^31^ The change from baseline in QoL score was assessed using a mixed model for repeated measures (MMRM); these longitudinal analyses were conducted by the treatment arm from baseline and for each assessment time point during 2 years after randomization. Longitudinal analyses were also repeated using the GVS score at inclusion (0 vs ≥ 1) in the olaparib-containing arm only.

## Results

### Study design and participants

**Figure 1.**
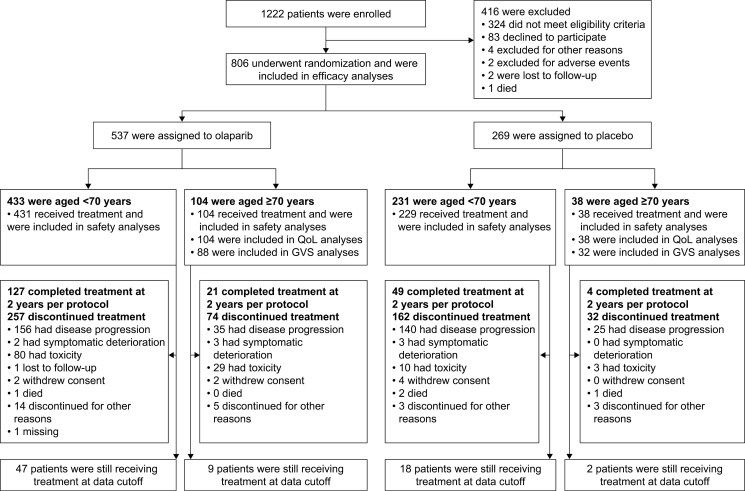
Study flow diagram. GVS, Geriatric Vulnerability Score; QoL, quality of life.

A total of 806 patients were randomized in PAOLA-1. Of these patients, 142 (17.6%) were ≥70 years old and 104 were randomized into the olaparib-containing arm (Figure 1).

### Clinical baseline characteristics of older patients included in both treatment arms

Age, ECOG PS, and HRD status were well-balanced between treatment arms in the older population (*n *= 142). However, compared with the placebo arm, there was a lower percentage of patients in the olaparib-containing arm with FIGO stage IV disease (27.9% vs 39.5%) and BRCAm (15.4% vs 21.1%). A higher percentage of patients in the olaparib-containing arm had upfront surgery (37.5% vs 28.9%) and upfront debulking surgery with macroscopic complete resection (19.2% vs 10.5%).

### Clinical characteristics and patterns of care description and comparison between older and younger patients in the olaparib-containing arm

The baseline clinical, pathological, and molecular characteristics of older (*n* = 104) and younger patients (*n *= 433) are summarized in Table 1. Median age was 73 years (range, 70-87 years) in older patients versus 59 years (range, 32-69 years) in younger patients. Older patients were less likely to undergo upfront debulking surgery with macroscopic complete resection (19.2% vs 32.3%, *P =.*009) or to have a BRCAm (15.4% vs 32.6%, *P* <.001) and an HRD-positive status (33.7% vs 50.8%, *P *<.001). Median olaparib plus bevacizumab treatment duration was shorter in older patients than in younger ones (median [minimum; maximum] of 14.9 months [0.0; 33.0] vs 17.9 [0.0; 32.1] months, respectively).

**Table 1.** Baseline characteristics in older (≥70 years old) and younger (<70 years old) patients.

Characteristic	Older patients (≥70 years old), *N* = 142		Younger patients (<70 years old), *N* = 664	
	Olaparib + bevacizumab (*n* = 104)	Placebo + bevacizumab (*n* = 38)	Olaparib + bevacizumab (*n* = 433)	Placebo + bevacizumab (*n *= 231)
Median age (range), years	73 (70–87)	73 (70–85)	59 (32–69)	58 (26–69)
FIGO stage				
III	75 (72.1)	23 (60.5)	303 (70.0)	163 (70.6)
IV	29 (27.9)	15 (39.5)	130 (30.0)	68 (29.4)
ECOG PS				
0	65 (62.5)	22 (57.9)	313 (72.3)	167 (72.3)
1	36 (34.6)	16 (42.1)	117 (27.0)	60 (26.0)
Missing	3 (2.9)	0 (0)	3 (<1)	4 (1.7)
Surgical status				
Upfront surgery	39 (37.5)	11 (28.9)	232 (53.6)	127 (55.0)
Complete resection	20 (19.2)	4 (10.5)	140 (32.3)	81 (35.1)
Interval surgery	49 (47.1)	15 (39.5)	179 (41.3)	95 (41.1)
Complete resection	34 (32.7)	9 (23.7)	129 (29.8)	66 (28.6)
No surgery	16 (15.4)	12 (31.6)	22 (5.1)	9 (3.9)
Upfront surgery with no residual macroscopic disease vs other	20 (19.2) vs 84 (80.8)	4 (10.5) vs 34 (89.5)	140 (32.3) vs 293 (67.7)	81 (35.1) vs 150 (64.9)
Deleterious tBRCAm^a^				
Yes	16 (15.4)	8 (21.1)	141 (32.6)	72 (31.2)
No	88 (84.6)	30 (78.9)	292 (67.4)	159 (68.8)
Tumor HRD status				
Positive^b^	35 (33.7)	11 (28.9)	220 (50.8)	121 (52.4)
Negative	54 (51.9)	18 (47.4)	138 (31.9)	67 (29.0)
Unknown^c^	15 (14.4)	9 (23.7)	75 (17.3)	43 (18.6)

Data are *n* (%) unless otherwise stated. The median time from the first cycle of chemotherapy to randomization was 6 months.

^a^Per-eCRF.

^b^Defined as a tBRCAm or a genomic instability score ≥ 42 (Myriad MyChoice HRD Plus assay [Myriad Genetic Laboratories, Inc., Salt Lake City, UT]).

^c^Defined as an inconclusive, missing, or failed test.

Abbreviations: ECOG PS, Eastern Cooperative Oncology Group performance status; eCRF, electronic case report form; FIGO, International Federation of Gynecology and Obstetrics; HRD, homologous recombination deficiency; tBRCAm, tumor *BRCA1/BRCA2* mutation.

### Geriatric covariates of older patients in both treatment arms at baseline

More than one-third of the older population had clinical criteria of malnutrition (28.8% in the olaparib-containing arm and 36.8% in the placebo arm), with hypoalbuminemia present in <5% of cases (Table 2). More than 20% (olaparib-containing arm) and 30% (placebo arm) of patients exhibited a significant loss of autonomy, predominantly detected in IADL. Few patients (<10% in both arms) had a CCI score of ≥ 5, but >40% of patients were taking antihypertensive medication. Additionally, 15%-18% of older patients had signs of depression and/or anxiety. A minority of patients were vulnerable (6 patients [5.8%] in the olaparib-containing arm and 3 patients [7.9%] in the placebo arm had a GVS score ≥ 3), but 46.5% of patients had at least 1 impairment on 1 of the 5 GVS items. The geriatric characteristics were well-balanced between both arms (Table 2).

**Table 2.** Baseline geriatric and biological/clinical parameters in older patients (≥70 years) included in the PAOLA-1 trial.

Geriatric parameters	Olaparib + bevacizumab (*n* = 104)	Placebo + bevacizumab (*n *= 38)
BMI, kg/m^2^		
<21	30 (28.8)	14 (36.8)
21–33	72 (69.2)	22 (57.9)
>33	1 (1.0)	2 (5.3)
Albumin <35 g/L	5 (4.8)	1 (2.6)
ADL scores		
ADL < 6	15 (14.4)	8 (21.1)
IADL < 25	23 (22.1)	12 (31.6)
Age-adjusted CCI		
3	39 (37.5)	15 (39.5)
4	61 (58.7)	20 (52.6)
5	4 (3.8)	3 (7.9)
Polypharmacy ≥5 drugs/day	1 (1)	0 (0)
Antihypertensive drug	44 (42.3)	18 (47.3)
HADS ≥ 14	16 (15.4)	7 (18.4)
**Hemoglobin <10 g/dL**	0 (0)	0 (0)
Lymphocyte count <1 G/L	14 (13.5)	2 (5.3)
**Neutrophil count <1.5 G/L**	1 (1.0)	0 (0)
**Platelet count <150 G/L**	9 (8.7)	8 (21.1)
Additional biological/clinical parameter		
Cytopenia^a^	24 (23.1)	9 (23.7)
**Renal function**		
Normal^b^	32 (30.8)	13 (34.2)
Mild and moderate insufficiency^c^	71 (68.3)	25 (65.8)
GVS score		
0	34 (32.7)	12 (31.6)
≥ 1	48 (46.2)	18 (47.4)
≥ 3	6 (5.8)	3 (7.9)

Data are *n* (% of data available) unless otherwise specified.

Items in bold were inclusion criteria in PAOLA-1: hemoglobin ≥10 g/dL, absolute neutrophil count ≥1.5 G/L, platelets ≥100 G/L, serum creatinine ≤1.25 × institutional upper limit of normal, and creatinine clearance >50 mL/min.

^a^Cytopenia included patients with anemia and/or leukopenia and/or neutropenia and/or lymphopenia and/or thrombocytopenia.

^b^Normal renal function was defined by creatinine clearance >90 mL/min (Cockcroft and Gault formula).

^c^Mild renal insufficiency was defined by creatinine clearance between 60 and 89 mL/min and moderate renal insufficiency by creatinine clearance between 30 and 59 mL/min.

Abbreviations: ADL, Activities of Daily Living; BMI, body mass index; CCI, Charlson Comorbidity Index; GVS, Geriatric Vulnerability Score; HADS, Hospital Anxiety and Depression Scale; IADL, Instrumental Activities of Daily Living.

### Olaparib safety profile compared with placebo in the older population

Older patients receiving olaparib (*n = *104), compared with placebo (*n *= 38), exhibited increased rates of all-grade anemia (48.1% vs 13.2%), neutropenia (22.1% vs 15.8%), lymphopenia (31.7% vs 13.2%), and thrombocytopenia (10.6% vs 2.6%), consistent with the AE profile of olaparib. Older patients receiving olaparib exhibited more frequent grade ≥3 anemia (21.2% vs 0%), but the frequency of grade ≥3 neutropenia and thrombocytopenia was similar in both arms (Supplementary Table S1). As any-grade lymphopenia was more frequent in the olaparib-containing arm, we explored baseline lymphocyte counts in both treatment arms. The lymphopenia rate at inclusion was slightly higher in the olaparib-containing arm than in the placebo arm (13.5% vs 5.3%); nevertheless, the incidence of lymphopenia increased to 31.7% with olaparib and 13.2% with placebo after treatment initiation, suggesting an association with olaparib treatment.

Concerning nonhematological AEs, older women treated with olaparib had more asthenia/fatigue (53.8% vs 31.6%), digestive disorders (including nausea [47.1% vs 18.4%], vomiting [23.1% vs 5.3%], and decreased appetite [11.5% vs 5.3%]), and abdominal pain (19.2% vs 13.2%) than patients receiving placebo. Rates of all-grade (53.8% vs 78.9%) and grade ≥3 (26.9% vs 39.5%) hypertension were lower in the olaparib-containing arm. No serious AEs of specific interest, such as myelodysplastic syndrome, acute leukemia (Supplementary Table S1), or treatment-related deaths, were reported during the treatment period.

### Comparison of olaparib safety between older and younger patients

Among the most common AEs, lymphopenia (all grades) and grade ≥3 hypertension were higher in older patients (*n *= 104) than in younger patients (*n *= 431) (31.7% vs 21.6%, *P* =.032 and 26.9% vs 16.7%, *P* =.019, respectively). There were no new AEs described specifically in the older population (Table 3).

**Table 3.** Safety profile of olaparib + bevacizumab treatment in older (≥70 years old; *n* = 104) and younger patients (<70 years old; *n *= 431) in the PAOLA-1 trial.

AEs	All grades		*P*-value	Grade ≥3		*P*-value
	≥70 years	<70 years		≥70 years	<70 years	
All	104 (100)	427 (99.1)		71 (68.3)	232 (53.8)	
Anemia^a^	50 (48.1)	169 (39.2)	.114	22 (21.2)	71 (16.5)	.276
Leukopenia^b^	20 (19.2)	75 (17.4)		2 (1.9)	8 (1.9)	
Neutropenia^c^	23 (22.1)	72 (16.7)	.210	10 (9.6)	22 (5.1)	.086
Lymphopenia^d^	33 (31.7)	93 (21.6)	.032	9 (8.7)	29 (6.7)	.509
Thrombocytopenia^e^	11 (10.6)	31 (7.2)		2 (1.9)	7 (1.6)	
Asthenia/fatigue	56 (53.8)	227 (52.7)		6 (5.8)	22 (5.1)	
Arthralgia	14 (13.5)	102 (23.7)		1 (1.0)	2 (0.5)	
Nausea	49 (47.1)	236 (54.8)	.135	0	13 (3.0)	.072
Hypertension	56 (53.8)	189 (43.9)	.079	28 (26.9)	72 (16.7)	.019
Proteinuria	6 (5.8)	25 (5.8)		0	5 (1.2)	
Myelodysplastic syndrome	0 (0)	3 (0.7)		0	3 (0.7)	
Acute leukemia	0 (0)	1 (0.2)		0	1 (0.2)	
Dose reduction	48 (46.2)	172 (39.9)		N/A	N/A	
Treatment interruption	62 (59.6)	22 (53.1)		N/A	N/A	
Treatment discontinuation	28 (26.9)	81 (18.8)		N/A	N/A	

Data are given as *n* (% of cases available) unless otherwise specified. AEs were graded according to the National Cancer Institute Common Terminology Criteria for Adverse Events, version 4.03.

^a^Grouped term including patients with anemia, decreased hemoglobin level, decreased hematocrit, and decreased red blood cell count.

^b^Patients with leukopenia or a decreased white blood cell count.

^c^Includes patients with neutropenia, febrile neutropenia, or agranulocytosis.

^d^Decreased lymphocyte count, lymphopenia, a decreased B-lymphocyte count, or a decreased T-cell count.

^e^Includes patients with thrombocytopenia—decreased platelet production.

Abbreviations: AE, adverse event; N/A, not applicable.

In the olaparib-containing arm, older patients experienced higher rates of grade ≥3 toxicity (68.3% vs 53.8%), toxicities leading to dose reductions (46.2% vs 39.9%), treatment interruptions (59.6% vs 53.1%), and treatment discontinuations (26.9% vs 18.8%) than their younger counterparts (Table 3). The main AEs that led to dose reduction and discontinuation were, in order of frequency, anemia (23.1% and 6.7%, respectively), nausea (6.7% and 3.8%, respectively), and asthenia/fatigue (2.9% and 1.9%, respectively). These were similar regardless of age.

### QoL with olaparib in older patients

#### Compliance with questionnaire completion

Compliance rates for QLQ-C30 and QLQ-OV28 were high at baseline and similar in each arm (100% in the olaparib-containing arm and 94.7% in the placebo arm; Supplementary Figure S1). The overall compliance rates over 96 weeks remained high (70.9% in the olaparib-containing arm and 72.0% in the placebo arm). At baseline, QoL scores were well-balanced between the treatment arms.

#### Global Health Status difference from baseline to the end of maintenance in the olaparib-containing arm versus the placebo arm

**Figure 2.**
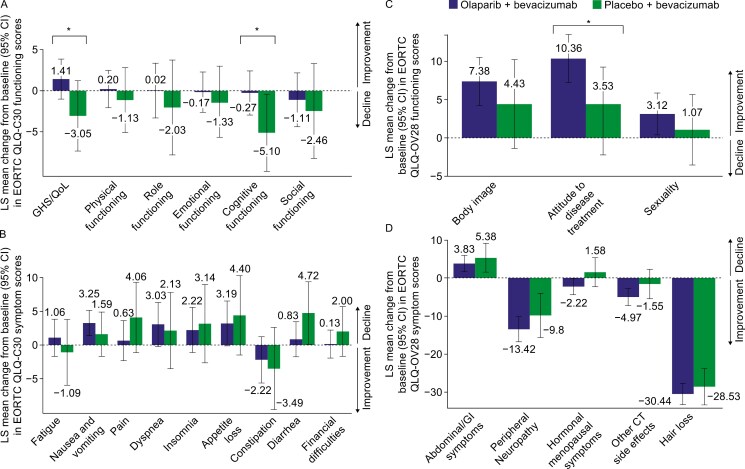
Least-squares mean change from baseline to month 24 of the functional (A) and symptomatic domain (B) EORTC QLQ-C30 scores and the functional (C) and symptomatic domain (D) EORTC QLQ-OV28 scores in older patients (≥70 years old). *Clinically significant difference, according to Musoro et al.^31^ CT, chemotherapy; EORTC QLQ-C30, European Organization for Research and Treatment of Cancer Quality of Life Questionnaire Core 30; EORTC QLQ-OV28, European Organization for Research and Treatment of Cancer Quality-of-Life Questionnaire-Ovarian 28; GHS, Global Health Status; GI, gastrointestinal; LS, least-squares; QoL, quality of life.

At the end of the 24-month treatment period, the mean GHS was numerically higher in the olaparib-containing arm (adjusted mean change from MMRM of +1.41 from baseline [95% CI, −1.01 to 3.84]) than in the placebo group (adjusted mean change of −3.05 from baseline [95% CI, −7.32 to 1.22]). The adjusted mean difference over 24 months was +4.47 points (95% CI, −0.49 to 9.42), reaching the minimally important difference of 4 points in favor of the olaparib-containing arm (Figure 2A).

#### Differences in functional, symptom-specific, and symptomatic scales from baseline to the end of maintenance in the olaparib-containing arm versus the placebo arm

Regarding the QLQ-C30 functional scales, only cognitive functioning indicated clinically relevant results between treatment arms following 24 months of treatment, with numerically lower deterioration in cognitive dimension in the olaparib-containing arm than in the placebo arm (difference: +4.82 [95% CI, −0.57 to 10.21]) (Figure 2A). Concerning QLQ-OV28 functional scales, attitude to disease and treatment was improved in both treatment arms but with a higher amplitude in the olaparib-containing arm, resulting in an adjusted mean difference of +6.82 (95% CI, 0.25-13.40) between treatment arms (Figure 2C). In contrast, body image and sexuality scales were similar between treatment arms (Figure 2C). The QLQ-C30 and QLQ-OV28 results did not show any significant difference in symptomatic scales between treatment arms (Figure 2B and D).

#### Association of the GVS with toxicity and QoL in the olaparib versus the placebo arm

In the olaparib-containing arm, older patients presenting with GVS ≥ 1 experienced more grade ≥3 AEs, serious AEs, AEs leading to dose reduction, and AEs leading to treatment interruption than older patients with a normal GVS. However, the rate of treatment discontinuation due to AEs was in the same range in both GVS groups (Table 4). These results tend to be similar in the placebo arm, where toxicity was worse in older patients with impaired GVS (Supplementary Table S2).

**Table 4.** Summary of AEs by GVS in older (≥70 years old) patients receiving olaparib plus bevacizumab.

AEs	GVS = 0 (*n *= 34)	GVS ≥ 1 (*n* = 48)
Grade ≥3	21 (61.8)	35 (72.9)
Serious AEs	12 (35.3)	21 (43.8)
Leading to dose interruption	17 (50.0)	32 (66.7)
Leading to dose reduction	12 (35.3)	26 (54.2)
Leading to discontinuation	10 (29.4)	13 (27.1)
Leading to death	0 (0)	0 (0)

Data are *n* (%) unless otherwise stated. Relationship to the indicated agent was assessed by the investigator. AEs were graded according to the CTCAE, version 4.03.

Abbreviations: AE, adverse event; CTCAE, National Cancer Institute Common Terminology Criteria for Adverse Events; GVS, Geriatric Vulnerability Score.

**Figure 3.**
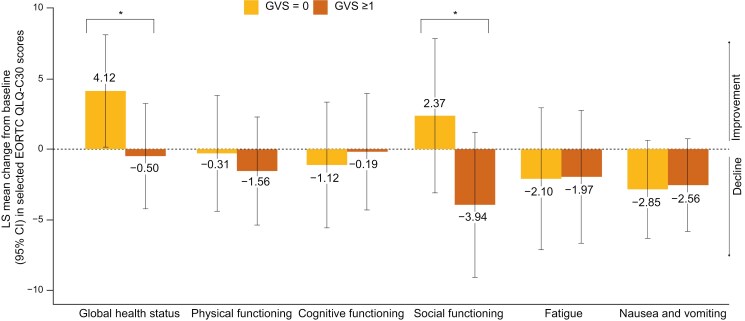
Least-squares mean longitudinal change from baseline to month 24 of selected EORTC QLQ-C30 scores in older patients (≥70 years old) receiving olaparib according to GVS group. Results from fatigue and nausea and vomiting scores have been reversed on the graph so that a positive change will correspond with an improvement. *Clinically significant difference, according to Musoro et al.^31^ EORTC QLQ-C30, European Organization for Research and Treatment of Cancer Quality of Life Questionnaire Core 30; GVS, Geriatric Vulnerability Score; LS, least-squares.

Considering the olaparib-bevacizumab arm, we observed that at baseline, older patients with a normal GVS had a better GHS and role, emotional, and cognitive QLQ-C30 scores than older patients with GVS ≥ 1 (Supplementary Table S3). Concerning QLQ-OV28 scores, similar results were observed for attitude to disease and body image (Supplementary Table S4). After 2 years of treatment, the GHS and social functioning QLQ-C30 scores seemed better in older patients with normal GVS than in patients with GVS ≥ 1 (Figure 3 and Supplementary Figure S2). Similarly, in the placebo arm, elderly patients with a normal GVS had better QoL values than those with a GVS value of ≥ 1 (Supplementary Tables S5 and S6 and Supplementary Figure S3).

## Discussion

In this preplanned exploratory analysis of PAOLA-1, we reported toxicity and QoL in older patients treated with olaparib versus placebo plus bevacizumab maintenance therapy and evaluated the impact of GVS on these criteria. The safety profile of older patients treated with olaparib plus bevacizumab was consistent with previously published data (mainly characterized by cytopenia, asthenia, and gastrointestinal AEs) but cases of all-grade lymphopenia and grade ≥3 hypertension on treatment were higher in older patients than in their younger counterparts. Following 24 months of treatment, patients receiving olaparib tended to have better GHS and cognitive functioning scores and similar results in other QoL items compared with the placebo arm. Older patients who exhibited GVS ≥ 1 at baseline who received olaparib treatment had increased toxicity and poorer QoL than those with a GVS value of 0.

Our analysis focused on older patients during the maintenance phase of treatment, with all patients having received chemotherapy and 85% of older patients in the olaparib-containing arm having undergone surgery. In addition, strict criteria for general condition (ECOG PS ≤ 1) and normal organ function were applied for patient selection. However, the impact of performance status on toxicity is currently debated in the literature.^32,33^ Although “hyperselected,” our population presented some criteria of frailty. For example, >30% of patients were malnourished, 14%-32% had impaired ADL and IADL scores, 16% had symptoms of anxiety and/or depression, 23% had cytopenia, and >45% had a GVS value of ≥ 1. In OC, hypoalbuminemia, lymphopenia, depression, and loss of autonomy have previously been associated with an increased risk of toxicity.^34,35^ In our study, these criteria were well-balanced between the treatment arms at baseline.

Anemia is the most common hematologic toxicity of PARP inhibitors. The frequencies of all-grade and grade ≥3 anemia reached, respectively, 48% and 21% in this analysis, higher than previously published in randomized trials on younger populations, which reported frequencies of all-grade anemia and grade ≥3 anemia events of 17%-44% and 5%-22%, respectively.^10,36-38^ Therefore, the frequency of anemia in our study appears to be high in older patients treated with olaparib plus bevacizumab. An increased frequency of anemia events (all grades) was reported by Selle et al.^13^ in their retrospective study of patients ≥70 years of age (*n *= 121) undergoing bevacizumab treatment. Thus, it is not surprising that the combination of olaparib and bevacizumab induces a slightly increased rate of anemia compared with that described for PARP inhibitors alone. Nevertheless, in our study, the frequency of anemia was not significantly different between age groups. Our data support the results of the only comparative study in older patients undergoing olaparib treatment (*n* = 398), in which Dockery et al.^39^ found similar rates of anemia (all grades and grade ≥3) in the recurrent setting before and after age 65 years.

Moreover, lymphopenia (all grades) was also indicated to be more frequent in the older population; this side effect has also been previously reported in elderly patients on PARP inhibitors.^40^

Asthenia is a frequently reported side effect of PARP inhibitor use. In the literature, up to 66% of patients treated with olaparib experience all-grade and up to 7% experience grade ≥3 asthenia/fatigue.^10,36-38^ Rates of asthenia in our study were similar.

Nausea is one of the most common side effects associated with olaparib administration. Data from randomized trials report nausea of all grades in 53%-76% of patients taking olaparib and grade ≥3 in 1%-3% of cases.^10,36-38^ Nausea rates were slightly lower in our older population receiving olaparib (47% all-grade nausea, no grade ≥3 nausea). A trend toward reduced rates of nausea was previously reported with niraparib^41^; however, retrospective studies dedicated to older patients reported a similar rate of nausea regardless of age.^39,42^ Thus, our results need to be confirmed by other prospective studies.

No treatment-related deaths, acute leukemia, or myelodysplastic syndrome were reported with olaparib in older patients in this study. Previous data from registration trials estimated the risk of developing a hematological malignancy which was between 1% and 2%.^10,37,38^ In their meta-analysis, the Young International Society of Geriatric Oncology presented subgroup data of patients >65 years receiving olaparib (*n* = 38) and concluded that there is no increase in the risk of myeloid hemopathy with advancing age, a conclusion that is shared by retrospective studies and real-life data.^39,42,43^

In this analysis, grade ≥3 hypertension rates were higher in older patients treated with olaparib plus bevacizumab than in younger patients. This AE may be related to bevacizumab; studies of older patients treated with bevacizumab have reported an increased risk of hypertension.^13,15^ Although high, the incidence of hypertension was lower in patients receiving olaparib plus bevacizumab than in patients receiving bevacizumab treatment alone. This “antihypertensive” effect of olaparib could be explained by its in vivo role on angiogenesis transcription factors and on the renin–angiotensin–aldosterone system.^44,45^

We observed a higher QoL questionnaire completion rate in our study than had previously been reported.^46^ In the olaparib-containing arm, minimal clinical benefits on GHS, cognitive function, and attitude to disease scores were recorded, probably linked to better control of the disease and delayed progression and therefore symptoms linked to the disease being less present. However, these results have clinical significance as they contribute to the understanding, acceptance, and compliance of patients with the care plan throughout treatment. None of the QLQ-C30 or QLQ-OV28 symptom scales were worsened by the addition of olaparib. Although difficult to compare because of the diversity of the scores used and the recent revision of the threshold for interpretation of QoL scores,^31^ none of the studies conducted on olaparib in the maintenance setting demonstrated a deterioration in QoL.^10,37,47-49^ Added to recent QoL data in the PAOLA-1 study presented at the 2022 American Society of Clinical Oncology (ASCO) Annual Meeting,^50^ our results suggest that QoL is maintained regardless of age.

To the best of our knowledge, ours is the first analysis investigating the impact of geriatric variables on QoL. The GVS was initially developed in a population receiving chemotherapy, and this is the first time this score has been used to provide data on patients under targeted therapy. Patients are usually considered vulnerable at a threshold of 3, but this threshold could not be used in this analysis because of the small number of patients. We chose to define the cutoff as 1 (see Methods section), and future studies dedicated to the geriatric population will be useful to better determine this cutoff. Our analysis suggests that older patients with a GVS value of ≥ 1 experienced increased toxicity and poorer QoL than those with a GVS value of 0. However, patients with a GVS value of ≥ 1 had a similar rate of discontinuation of treatment to those with a normal GVS, suggesting that toxicity remained manageable with more dose reductions or interruption of treatment.

PAOLA-1 is one of the first international phase III studies on PARP inhibitors in which geriatric safety and QoL are considered. Methodologically, it is an a priori-defined subgroup analysis with prospective data collection. Although there was no upper age limit at inclusion, the fact that randomization took place at the end of a heavy therapeutic sequence led to a selection bias, which explains the low number of vulnerable patients. Results cannot be generalized to the whole elderly population but can be used to generate hypotheses, paving the way for future studies focused on this population in a real-world setting. All comorbidities and co-medications, excluding arterial hypertension and antihypertensive treatments, were not prospectively collected. Moreover, data concerning QoL were not compared with data from younger patients; further analyses dedicated to QoL in the whole PAOLA-1 population are warranted. Finally, no efficacy analysis in this older population was performed because of the imbalanced initial characteristics (FIGO stage, surgical treatment, and BRCAm frequency) between treatment arms in this population.

## Conclusion

In PAOLA-1, older patients treated with olaparib plus bevacizumab had a manageable safety profile similar to that observed in younger patients. GHS, functional, and symptom-specific scores seemed to be better 24 months after randomization in the olaparib-containing arm. These results support the use of olaparib and bevacizumab combination maintenance therapy in older patients with newly diagnosed advanced OC. Further analyses are required to confirm these results in unselected patients, notably the impact of geriatric covariates on safety and QoL in this setting.

## Supplementary Material

oyae322_suppl_Supplementary_Material

## Data Availability

ARCAGY-GINECO has a long history of academic data sharing for research purposes. The process is similar for every trial sponsored by ARCAGY-GINECO (or ARCAGY Research): • Researchers have to submit a request to the sponsor directly or through the principal investigator. The request should be written in a predefined format of a short synopsis indicating the objective of the research, the methodology intended to be used, including the statistical analysis plan, and the variables within the database required for the research. • A scientific board will review and approve the requests on a case-by-case basis. Only encoded datasets will be used, which enables us to fulfill legal and ethical obligations to protect our patients while at the same time utilizing patient data in progressing medical research to its full potential in the best interests of public health. A specific agreement between the sponsor and the researcher is requested for data transfer. This data transfer agreement details both parts responsibilities to ensure the required level of data integrity and legal and ethical obligations. In the case of sharing encoded patient level data, please note that the full dataset may not be shared in view of the following: • Clinical consent for some countries prohibits secondary use of the data. • Patients may withdraw their consent for participation in the trial at any point. • Other aspects might also be taken into consideration to protect patient privacy (eg, review of rare clinical events where information is aggregated to a higher level before sharing).
