# Supplementary material for: Safety and quality of life with maintenance olaparib plus bevacizumab in older patients with ovarian cancer: subgroup analysis of PAOLA‑1/ENGOT-ov25
Source: Oncologist. 2024 Dec 14;30(7):oyae322. doi: 10.1093/oncolo/oyae322 (PMC12311279; doi:10.1093/oncolo/oyae322)
Supplement: oyae322_suppl_Supplementary_Material [file oyae322_suppl_supplementary_material.pdf]

Supplementary Material

Safety and Quality of Life with Maintenance Olaparib Plus Bevacizumab in Older Patients with Ovarian Cancer: Subgroup Analysis of PAOLA-1/ENGOT-OV25

Montégut C, Falandry C, Cinieri S *et al*

This appendix has been provided by the authors to give readers additional information about this work.

Supplementary Table S1. Safety in older patients (≥70 years old) between treatment arms.

AEs	All grades		Grade ≥3	
	**Olaparib + bevacizumab (*n* = 104)**	**Placebo + bevacizumab (*n* = 38)**	**Olaparib + bevacizumab (*n* = 104)**	**Placebo + bevacizumab (*n* = 38)**
All	104 (100)	38 (100)	71 (68.3)	23 (60.5)
Anemia[a]	50 (48.1)	5 (13.2)	22 (21.2)	0 (0)
Leukopenia[b]	20 (19.2)	1 (2.6)	2 (1.9)	1 (2.6)
Neutropenia[c]	23 (22.1)	6 (15.8)	10 (9.6)	4 (10.5)
Lymphopenia[d]	33 (31.7)	5 (13.2)	9 (8.7)	1 (2.6)
Thrombocytopenia[e]	11 (10.6)	1 (2.6)	2 (1.9)	1 (2.6)
Asthenia/fatigue	56 (53.8)	12 (31.6)	6 (5.8)	1 (2.6)
Nausea	49 (47.1)	7 (18.4)	0 (0)	1 (2.6)
Vomiting	24 (23.1)	2 (5.3)	1 (1.0)	0 (0)
Diarrhea	21 (20.2)	6 (15.8)	3 (2.9)	1 (2.6)
Decreased appetite	12 (11.5)	2 (5.3)	0 (0)	1 (2.6)
Abdominal pain	20 (19.2)	5 (13.2)	1 (1.0)	1 (2.6)
Hypertension	56 (53.8)	30 (78.9)	28 (26.9)	15 (39.5)
Proteinuria	6 (5.8)	4 (10.5)	0 (0)	0 (0)
Myelodysplasia	0 (0)	0 (0)	0 (0)	0 (0)
Acute leukemia	0 (0)	0 (0)	0 (0)	0 (0)
Dose reduction	48 (46.2)	5 (13.2)	N/A	N/A
Treatment interruption	62 (59.6)	10 (26.3)	N/A	N/A
Treatment discontinuation	28 (26.9)	4 (10.5)	N/A	N/A

Data are given as *n* (% of cases available) unless otherwise specified. AEs were graded according to the National Cancer Institute Common Terminology Criteria for Adverse Events, version 4.03.

[a]Grouped term including patients with anemia, decreased hemoglobin level, decreased hematocrit, and decreased red blood cell count.

[b]Patients with leukopenia or a decreased white blood cell count.

[c]Includes patients with neutropenia, febrile neutropenia, or agranulocytosis.

[d]Decreased lymphocyte count, lymphopenia, a decreased B-lymphocyte count, or a decreased T-cell count.

[e]Includes patients with thrombocytopenia or decreased platelet production.

Abbreviations: AE, adverse event; N/A, not applicable.

Supplementary Table S2. Summary of AEs by GVS in older (≥70 years old) patients receiving placebo plus bevacizumab.

**AEs, n (%)**	**GVS = 0 (*n* = 12)**	**GVS ≥1 (*n* = 18)**
Grade ≥3	6 (50.0)	12 (66.7)
Serious AEs	3 (25.0)	10 (55.6)
Leading to dose interruption	4 (33.3)	6 (33.3)
Leading to dose reduction	1 (8.3)	4 (22.2)
Leading to discontinuation	1 (8.3)	2 (11.1)
Leading to death	0 (0.0)	0 (0.0)

AEs were graded according to the CTCAE, version 4.03.

Abbreviations: AE, adverse event; CTCAE, National Cancer Institute Common Terminology Criteria for Adverse Events; GVS, Geriatric Vulnerability Score.

Supplementary Table S3. Baseline health-related QoL in EORTC QLQ-C30 scores according to the GVS score in the olaparib-containing arm.

QLQ-C30 questionnaire	GVS	
	**0 (*n* = 34)**	**≥1 (*n* = 40)**
GHS		
N	33	39
Mean (SD)	75.8 (16.3)	63.9 (18.1)
Physical		
N	34	40
Mean (SD)	80.4 (16.3)	76.9 (18.7)
Role		
N	34	40
Mean (SD)	84.8 (20.3)	71.7 (32.3)
Emotional		
N	34	40
Mean (SD)	83.8 (13.9)	75.2 (23.5)
Cognitive		
N	34	40
Mean (SD)	87.7 (16.1)	77.9 (25.4)
Social		
N	34	40
Mean (SD)	80.9 (19.7)	78.8 (27.0)
Fatigue[a]		
N	34	40
Mean (SD)	28.3 (17.6)	38.6 (25.3)
Nausea and vomiting[a]		
N	34	40
Mean (SD)	4.4 (13.2)	7.1 (14.6)
Pain[a]		
N	34	40
Mean (SD)	17.6 (22.1)	16.7 (17.7)
Dyspnea[a]		
N	34	39
Mean (SD)	17.6 (22.1)	18.8 (22.7)
Insomnia[a]		
N	34	40
Mean (SD)	15.7 (24.9)	27.5 (31.0)
Appetite loss[a]		
N	34	40
Mean (SD)	4.9 (14.5)	10.8 (23.1)
Constipation[a]		
N	34	40
Mean (SD)	21.6 (30.6)	22.5 (24.3)
Diarrhea[a]		
N	34	39
Mean (SD)	10.8 (21.3)	10.3 (20.5)
Financial difficulties[a]		
N	33	40
Mean (SD)	2.0 (8.1)	2.5 (8.9)

[a]Symptomatic scale – a high score represents a high symptomatic level. For all other scales, a high score represents a high QoL level.

Abbreviations: EORTC QLQ-C30, European Organisation for Research and Treatment of Cancer Quality of Life Questionnaire Core 30; GHS, Global Health Status; GVS, Geriatric Vulnerability Score; QoL, quality of life; SD, standard deviation.

Supplementary Table S4. Baseline health-related EORTC QLQ-OV28 scores according to GVS score in the olaparib-containing arm.

**QLQ-OV28 questionnaire**	**GVS**	
	**0 (*n* = 34)**	**≥1 (*n* = 40)**
Body image		
N	34	40
Mean (SD)	79.4 (19.7)	62.5 (32.6)
Sexuality		
N	33	36
Mean (SD)	6.3 (18.6)	2.5 (10.3)
Attitude to disease/treatment		
N	34	40
Mean (SD)	53.6 (26.3)	43.2 (32.6)
GI/abdominal symptoms[a]		
N	34	40
Mean (SD)	15.7 (18.2)	15.6 (14.4)
Peripheral neuropathy[a]		
N	34	40
Mean (SD)	44.6 (34.3)	49.4 (30.4)
Hormonal/menopausal symptoms[a]		
N	34	40
Mean (SD)	8.3 (13.8)	11.2 (19.0)
Other chemotherapy side effects[a]		
N	34	40
Mean (SD)	25.0 (16.7)	29.8 (20.4)
Hair loss[a]		
N	32	39
Mean (SD)	43.2 (40.4)	37.6 (37.6)

[a]Symptomatic scale – a high score represents a high symptomatic level. For all other scales, a high score represents a high QoL level.

Abbreviations: EORTC QLQ-OV28, European Organisation for Research and Treatment of Cancer Quality-of-Life Questionnaire-Ovarian 28; GI, gastrointestinal; GVS, Geriatric Vulnerability Score; QoL, quality of life; SD, standard deviation.

Supplementary Table S5. Baseline health-related QoL in EORTC QLQ-C30 scores according to GVS score in the placebo-containing arm.

**QLQ-C30 questionnaire**	**GVS**	
	**0 (*n* = 11)**	**≥1 (*n* = 13)**
GHS		
N	11	12
Mean (SD)	68.9 (18.1)	54.9 (13.5)
Physical		
N	11	13
Mean (SD)	82.3 (17.9)	59.1 (19.5)
Role		
N	11	13
Mean (SD)	74.2 (29.2)	62.8 (27.3)
Emotional		
N	11	12
Mean (SD)	81.1 (17.9)	65.3 (26.6)
Cognitive		
N	11	12
Mean (SD)	89.4 (17.1)	76.4 (16.6)
Social		
N	11	12
Mean (SD)	83.3 (14.9)	68.1 (24.1)
Fatigue[a]		
N	11	13
Mean (SD)	34.3 (20.8)	50.9 (19.8)
Nausea and vomiting[a]		
N	11	13
Mean (SD)	3.0 (10.1)	5.1 (10.5)
Pain[a]		
N	11	13
Mean (SD)	31.8 (25.2)	28.2 (23.0)
Dyspnea[a]		
N	11	13
Mean (SD)	6.1 (13.5)	25.6 (27.7)
Insomnia[a]		
N	11	13
Mean (SD)	15.2 (22.9)	35.9 (31.8)
Appetite loss[a]		
N	11	13
Mean (SD)	9.1 (21.6)	33.3 (30.4)
Constipation[a]		
N	11	13
Mean (SD)	30.3 (37.9)	28.2 (35.6)
Diarrhea[a]		
N	11	12
Mean (SD)	12.1 (27.0)	11.1 (21.7)
Financial difficulties[a]		
N	11	12
Mean (SD)	0.0 (0.0)	13.9 (17.2)

[a]Symptomatic scale – a high score represents a high symptomatic level. For all other scales, a high score represents a high QoL level.

Abbreviations: EORTC QLQ-C30, European Organisation for Research and Treatment of Cancer Quality of Life Questionnaire Core 30; GHS, Global Health Status; GVS, Geriatric Vulnerability Score; QoL, quality of life; SD, standard deviation.

Supplementary Table S6. Baseline health-related QoL in EORTC QLQ-OV28 scores according to the GVS score in the placebo-containing arm.

**QLQ-OV28 questionnaire**	**GVS**	
	**0 (*n* = 11)**	**≥1 (*n* = 13)**
Body image		
N	10	13
Mean (SD)	71.7 (32.4)	56.4 (21.0)
Sexuality		
N	10	13
Mean (SD)	7.5 (15.9)	7.1 (12.7)
Attitude to disease/treatment		
N	11	13
Mean (SD)	47.5 (20.5)	33.3 (29.0)
GI/abdominal symptoms[a]		
N	10	13
Mean (SD)	26.2 (19.7)	23.4 (14.9)
Peripheral neuropathy[a]		
N	10	13
Mean (SD)	61.1 (29.3)	65.8 (28.9)
Hormonal/menopausal symptoms[a]		
N	10	13
Mean (SD)	1.7 (5.3)	10.3 (12.8)
Other chemotherapy side effects[a]		
N	10	13
Mean (SD)	24.7 (18.1)	31.3 (22.2)
Hair loss[a]		
N	10	13
Mean (SD)	28.3 (38.5)	47.4 (48.0)

[a]Symptomatic scale – a high score represents a high symptomatic level. For all other scales, a high score represents a high QoL level.

Abbreviations: EORTC QLQ-OV28, European Organisation for Research and Treatment of Cancer Quality-of-Life Questionnaire-Ovarian 28; GI, gastrointestinal; GVS, Geriatric Vulnerability Score; QoL, quality of life; SD, standard deviation.

Supplementary Figure S1. EORTC QLQ-C30 and EORTC QLQ-OV28 questionnaire completion.

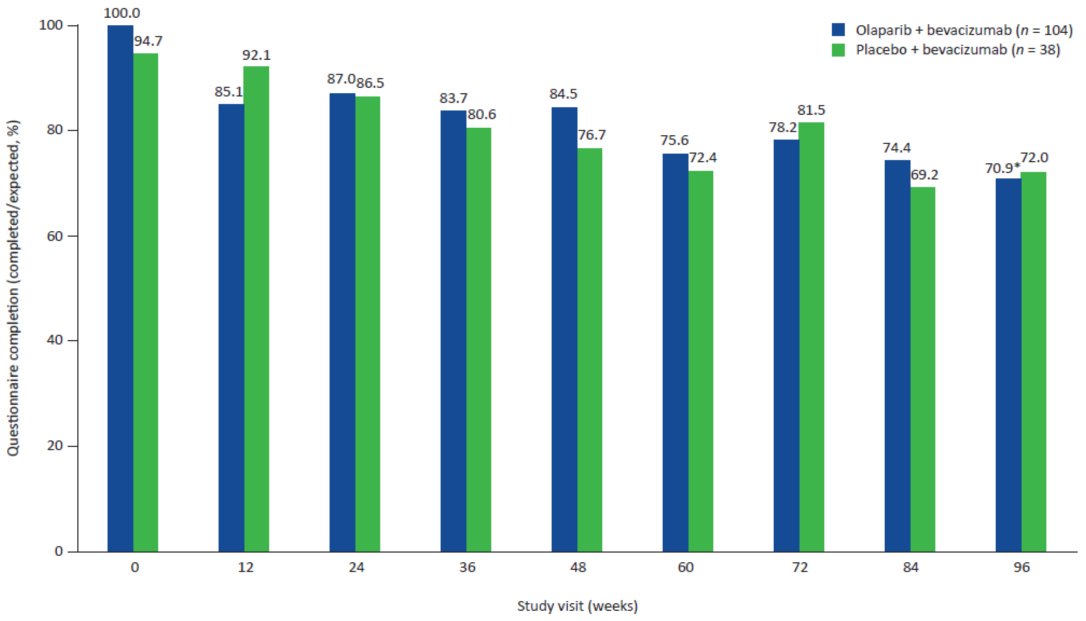

Percentages from the combined number of QLQ-C30 and QLQ-OV28 questionnaires completed/expected at the given time point, except where noted.

*71.4% for QLQ-OV28.

Abbreviations: EORTC QLQ-C30, European Organisation for Research and Treatment of Cancer Quality of Life Questionnaire Core 30; EORTC QLQ-OV28, European Organisation for Research and Treatment of Cancer Quality-of-Life Questionnaire-Ovarian 28.

Supplementary Figure S2. Least-squares mean longitudinal change from baseline to month 24 of all EORTC QLC-C30 scores in older patients (≥70 years old) receiving olaparib according to GVS group.

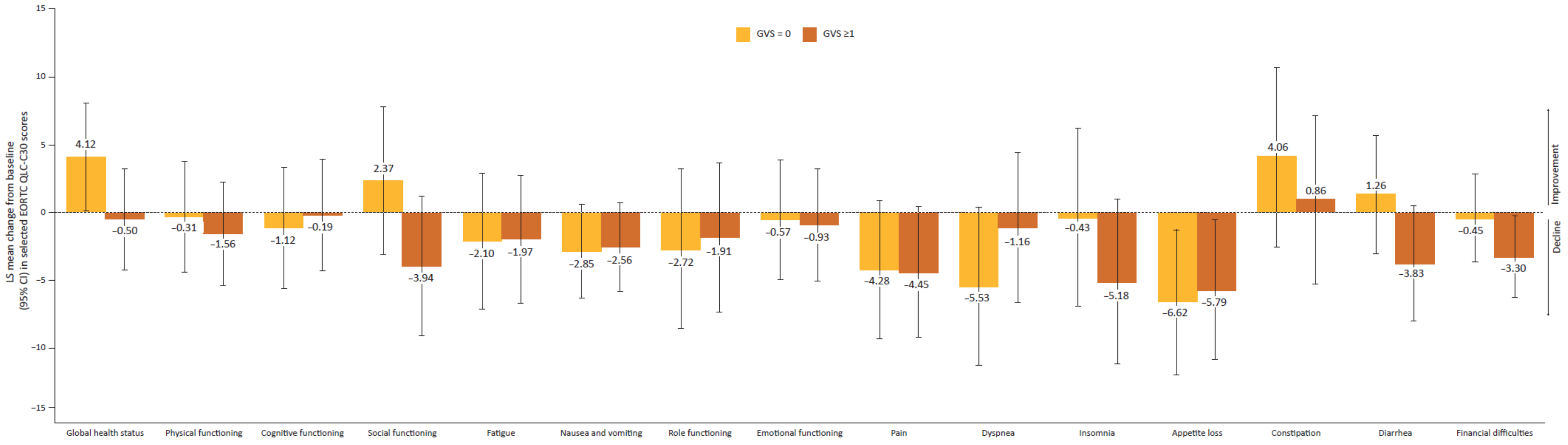

Results from all symptom scores have been reversed on the graph so that a positive change will correspond with an improvement.

Abbreviations: CI, confidence interval; EORTC QLQ-C30, European Organisation for Research and Treatment of Cancer Quality of Life Questionnaire Core 30; GVS, Geriatric Vulnerability Score; LS, least-squares.

Supplementary Figure S3. Least-squares mean longitudinal change from baseline to month 24 of selected EORTC QLC-C30 scores in older patients (≥70 years old) receiving placebo according to GVS group.

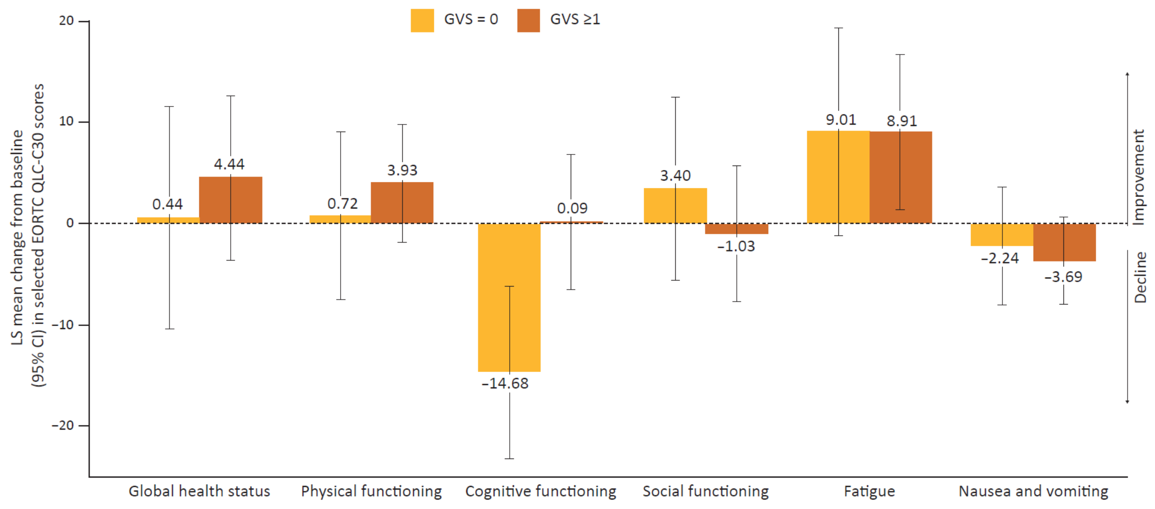

Results from fatigue and nausea and vomiting scores have been reversed on the graph so that a positive change will correspond with an improvement.

Abbreviations: CI, confidence interval; EORTC QLQ-C30, European Organisation for Research and Treatment of Cancer Quality of Life Questionnaire Core 30; GVS, Geriatric Vulnerability Score; LS, least-squares.
